# A pilot investigation of emotional regulation difficulties and mindfulness-based strategies in manic and remitted bipolar I disorder and major depressive disorder

**DOI:** 10.1186/s40345-020-00206-0

**Published:** 2021-01-04

**Authors:** Christie W. Musket, Natasha S. Hansen, Keith M. Welker, Kirsten E. Gilbert, June Gruber

**Affiliations:** 1 Department of Psychology, University of Pittsburgh, Pittsburgh, USA; 2 Department of Psychology and Neuroscience, University of Colorado, Boulder, 345 UCB, Muenzinger D321C, Boulder, CO 80309-0345 USA; 3 Department of Psychology, University of Massachusetts, Boston, USA; 4 Department of Psychiatry, Washington University in St. Louis, St. Louis, USA

**Keywords:** Emotion regulation, Emotional awareness, Impulsivity, Mindfulness, Bipolar disorder, Major depressive disorder

## Abstract

**Background:**

Both bipolar disorder and major depressive disorder are characterized by difficulties in emotion regulation. Little is known about which specific emotion regulatory patterns may be transdiagnostic versus disorder specific, and how such patterns change as a function of current mood states.

**Methods:**

This preliminary investigation examined specific patterns of self-reported trait emotion regulation difficulties and mindfulness-based regulations strategies across four groups: remitted adults with bipolar I disorder (BD-remitted; *n* = 32), currently manic adults with bipolar I disorder (BD-manic; *n* = 19), remitted adults with major depressive disorder (MDD-remitted; *n* = 32), and healthy controls (CTL; *n* = 30).

**Results:**

All three clinical groups reported significantly greater difficulties with emotion regulation and decreased overall mindfulness-based strategies.

**Conclusions:**

These results suggest that increased emotion regulation difficulties, decreased mindfulness, and increased emotion-driven impulsivity may be transdiagnostic across mood disorders and states, and that impulsivity may be particularly impaired during periods of mania.

## Background

Emotion regulation is defined as the set of actions that individuals take (either consciously or unconsciously) to affect their emotional experience (Gross and Jazaieri 2014). Emotion regulation can be either adaptive or maladaptive (i.e., can either improve or worsen individuals’ functioning or internal experience). An expanding literature highlights the prevalence and severity of emotion regulation difficulties across many different psychopathologies, including major depression and bipolar disorder (Gross and Jazaieri 2014; Aldao et al. 2010). In spite of the prevalence of such difficulties, there is a lack of research examining the similarities and differences in specific aspects of emotion dysregulation within and across mood disorders.

Bipolar disorder (BD) and major depressive disorder (MDD) are severe and chronic mood disorders. Disruptions in emotion regulation have been proposed as critical predictors of the etiology and maintenance of both BD and MDD (e.g., Alloy et al. 2009; Gruber 2011). However, less is known about whether emotion regulation patterns are stable across time or vary as a function of current mood state. For bipolar disorder in particular, there is a dearth of research exploring the patterns of emotion dysregulation in manic states. Furthermore, it is not known if such regulatory patterns are transdiagnostic across mood disorders. The goal of this pilot study was to begin to examine self-reported emotion regulation patterns across bipolar and unipolar mood disorders.

### Emotion regulation and mood disorders

BD is centrally characterized not only by extreme emotion fluctuation but also by impaired emotion regulation (Gruber 2011). Individuals with remitted BD tend to use more maladaptive emotion regulation strategies known to amplify emotional distress, including suppression and rumination (Gruber et al. 2013; Wolkenstein et al. 2014). Remitted BD individuals also tend to engage in maladaptive emotion-related impulsivity, which is associated with increased manic symptom severity and poorer global functioning (e.g., (Muhtadie et al. 2014). These findings suggest that problematic emotion regulation is a central feature of BD. However, little research has been conducted to determine whether or not such findings are state-specific (i.e., persist across mood phases in BD). Emotion regulation deficits are also a central feature of MDD (Rottenberg et al. 2005). Individuals with remitted MDD use more maladaptive emotion regulation strategies compared to individuals without any psychiatric diagnoses (Garnefski and Kraaij 2006; Joormann 2010), and have a tendency to up-regulate negative emotion intensity and fail to maintain positive emotion intensity (Gruber et al.. 2014). It still remains unclear whether or not such features are disorder-specific to unipolar depression or transdiagnostic across mood disorders.

One concept central to emotion regulation is mindfulness. Mindfulness is a mental state defined by bringing awareness to the present moment and accepting thoughts and feelings as they arise without immediate reaction or judgment (e.g., Kabat-Zinn 1990). Mindfulness has been shown to facilitate adaptive emotion regulation, and to promote physical and psychological well being (e.g., Kang et al. 2013). Recent work suggests that mindfulness-based emotion regulation strategies may help reduce mood symptom severity in both BD and MDD (Deckersbach et al. 2012; Teasdale et al. 2000; Hanssen et al. 2019). Individuals with BD even appear to benefit emotionally and physiologically from engaging in mindfulness-based instructions in laboratory studies (Gilbert et al. 2013). One key aspect of mindfulness as it relates to the process of regulating emotions is emotional awareness, or the extent to which an individual is conscious of his/her present emotional state. The ability to notice and accurately distinguish between different emotions has been proposed as a first step in regulating emotions adaptively (Vine and Aldao 2014). Despite its probable role in adaptive emotion regulation, emotional awareness remains largely unexplored in BD and MDD. Moreover, although some preliminary work has found that those with BD and MDD may not differ from controls in their self-reported awareness of emotional states (Rheenen et al. 2015), other research has suggested that deficits in identifying and differentiating between emotions are a transdiagnostic feature of mood disorders (Vine and Aldao 2014).

The present study examined self-reported patterns of trait difficulties with emotion regulation, and mindfulness-based emotion regulation strategies across four groups: currently hypomanic/manic BD I (BD-manic), remitted BD I (BD-remitted), remitted MDD (MDD-remitted), and healthy non-psychiatric control (CTL) adults. Aim 1 examined group differences in trait emotion regulation difficulties. Based on prior research indicating that both BD and MDD are characterized by difficulties with emotion regulation (Gruber 2011; Rottenberg et al. 2005; Rheenen et al. 2015; Giovanelli et al. 2013), we predicted that all three clinical groups would exhibit greater trait emotion regulation difficulties compared to the CTL group. Aim 2 examined group differences in trait mindfulness-based regulation strategies. For Aim 2, we examined two competing perspectives: The first is that there should be decreased mindfulness-based strategies across remitted and symptomatic phases of BD and MDD as compared to healthy controls, based on work suggesting that mood disorders are characterized by difficulties in emotion differentiation (e.g., Vine and Aldao 2014), and that increasing mindfulness reduces both manic and depressive mood symptoms (Deckersbach et al. 2012; Teasdale et al. 2000). The second perspective posits intact emotion awareness (one aspect of mindfulness) across remitted and symptomatic phases in both BD and MDD based on research that suggests no significant deficit in self-reported emotional awareness in BD and MDD (Das et al. 2014; Ehring et al. 2008).

## Method

### Participants

Participants between the ages of 18–60 were recruited via posted flyers or online advertisements (e.g., www.craigslist.org) from the greater New Haven, CT area. Participants responded to one of three separate study advertisements: a study on “emotion and mood” for healthy controls, on “bipolar disorder and emotion” for the BD group, and on “history of depression and emotion” for the MDD group. Interested participants completed a brief phone screen with a trained researcher and were invited to the laboratory for a diagnostic evaluation to determine final study eligibility (see below).

The study sample consisted of four groups of individuals who met the following DSM-IV criteria: currently remitted BD type I (BD-remitted; *n* = 32), currently manic BD type I (BD-manic, *n* = 19), currently remitted MDD (MDD; *n* = 32), and healthy controls with no current or past Axis I disorders (CTL; *n* = 30). Exclusion criteria included a lifetime history of neurological disease, severe head trauma, stroke, autoimmune disorder, severe medical illness, and alcohol or substance abuse in the past six months. Given that both BD and MDD are highly comorbid with other disorders, participants in the three clinical groups were not excluded based on comorbidities other than substance abuse or dependence within the past 6 months. Demographic and clinical data are presented in Table 1.

**Table 1:** Demographic and clinical characteristics

	BD-remitted (*n* = 32)	BD-manic (n = 19)	MDD-remitted (*n* = 32)	CTL (*n* = 30)
Demographic characteristics				
Age (yrs)	29.30 (8.80)	35.93 (13.00)	31.23 (11.38)	31.93 (9.36)
Female	65.60%	57.90%	64.50%	63.3%
Caucasian	90.60%	100%	90.60%	90.00%
Education (Yrs)	14.95 (2.39)	14.67 (2.38)	15.13 (2.26)	15.95 (2.37)
Employment status				
Full-time	12.50%	21.10%	18.80%	36.70%
Part-time	34.40%	15.80%	31.30%	30.00%
Unemployed (not student)	15.60%	47.40%	21.90%	16.70%
Unemployed (student)	37.50%	5.30%	28.10%	13.30%
Marital status				
Single (no relationship)	56.30%	15.80%	37.50%	40.00%
Single (in relationship)	9.40%	42.10%	28.10%	33.30%
Live-in partner	3.10%	0%	3.10%	6.70%
Married	28.10%	15.80%	12.50%	16.70%
Divorced or widowed	3.10%	26.30%	18.80%	3.30%
Annual income				
< $10 K	18.80%	47.40%	15.60%	13.30%
$10 K–$25 K	18.80%	15.80%	18.80%	10.00%
$26 K–$50 K	31.30%	21.10%	40.60%	26.70%
$51 K–$75 K	3.10%	10.50%	12.50%	20.00%
$76 K–$100 K	15.60%	5.30%	3.10%	16.70%
> $100 K	12.50%	0%	9.40%	13.30%
Clinical characteristics				
YMRS	1.88 (1.90)	15.00 (5.48)	1.72 (1.87)	1.17 (1.05)
IDS-C	4.22 (3.27)	17.68 (8.45)	5.03 (2.79)	2.00 (1.98)
GAF	75.78 (5.91)	61.42 (8.64)	79.03 (6.82)	88.03 (3.03)
# Comorbid disorders	0.50 (0.84)	1.63 (1.54)	0.78 (0.94)	–
# Medications	2.03 (1.52)	1.65 (1.11)	0.53 (0.84)	–
Age at depression onset (Yrs)	16.57 (7.08)	13.32 (4.62)	16.09 (7.26)	–
Depression duration (Yrs)	14.29 (10.07)	22.92 (15.39)	15.34 (10.36)	–
# Depressive episodes	14.87 (23.28)	9.50 (17.19)	5.47 (7.35)	–
Age at Mania Onset (Yrs)	19.72 (6.74)	17.13 (9.28)	–	–
Mania duration (Yrs)	11.13 (9.86)	19.97 (15.96)	–	–
# Manic episodes	12.33 (25.55)	46.45 (40.73)	–	–

*BD-remitted* Bipolar I disorder remitted group, *BD-manic* Bipolar I disorder manic group, *MDD* Major depressive disorder remitted group, *CTL* Healthy control group, *YMRS* Young Mania Rating Scale, *IDS-C* Inventory to Diagnose Depression, *GAF* Global Assessment of Functioning, *Age at Onset* Age of first depressive or manic episode, ^*#*^*Comorbid Disorders* the number of current DSM-IV-TR Axis I comorbidities, Mean values are displayed with standard deviations in parentheses where applicable

### Measures of clinical functioning

#### Diagnostic evaluation

Diagnoses were established using the Structured Clinical Interview for DSM-IV (SCID-IV; First et al. 2007), administered by a licensed clinical psychologist, trained clinical psychology doctoral student, or trained post-baccalaureate fellow. Illness duration, age of onset, and lifetime number of depressive and manic mood episodes were also obtained. Following common practices in inter-rater reliability in our lab (e.g., Gruber and Weinstock 2018; Ong et al. 2017), a large subset (*n* = 91; 81%) of videotaped interviews was reviewed by a second rater to evaluate the reliability of diagnostic ratings. These ratings were discussed at a group consensus meeting and scoring adjustments were made as necessary. Final ratings between the interviewer and consensus rater matched 100% (κ = 1.00) of primary diagnoses.

#### Mood symptoms

Current symptoms of depression were measured using the Inventory of Depressive Symptomatology (IDS-C; Trivedi et al. 2004), a 30-item clinician-rated assessment with scores ranging from 0 to 84, with higher scores indicating more depressive symptomatology. Current symptoms of mania were measured using the Young Mania Rating Scale (YMRS; (Young et al. 1978), an 11-item clinician-rated scale with scores ranging from 0 to 60, with higher scores indicating more manic symptomatology. Remitted status for the BD-remitted, MDD-remitted, and CTL groups was confirmed using the SCID-IV current mood disorder module criteria and scores below standardized cutoffs on the IDS-C (≤ 11) and YMRS (≤ 7). Current manic status for the BD-manic group was confirmed using SCID-IV criteria for a current manic episode (*n* = 16) or hypomanic episode (*n* = 3)^1^ in the past month, as well scores above the YMRS cutoff (> 7) in the past week. Intra-class correlations (ICCs) for the same subset of participants were strong for the YMRS (ICC = 0.98) and IDS-C (ICC = 0.97).

#### Global functioning

Participants’ occupational, social, and psychological functioning within the past week was assessed using the Global Assessment of Functioning scale (GAF; *DSM-IV Axis V*), which ranges from 1 (lowest level of functioning) to 100 (highest level of functioning). GAF ICCs for a large subset of study participants (*n* = 91) was high (= 0.86).

#### Difficulties in emotion regulation scale

The Difficulties in Emotion Regulation Scale (DERS; Gratz and Roemer 2004) is a 36-item questionnaire that provides an assessment of self-reported emotion regulation difficulties. Items (e.g., “When I'm upset, I have difficulty getting work done,” “I have difficulty making sense out of my feelings”) were rated using a 5-point Likert scale (1 = almost never, 5 = almost always). The DERS is made up of six subscales: Non-acceptance (i.e., a tendency to pass negative judgment on one’s own distress); Goals (i.e., trouble accomplishing goals while upset); Strategies (i.e., limited access to emotion regulation strategies); Impulse (i.e., the tendency to lose control of behaviors when emotionally upset); Awareness (i.e., attentiveness to one’s emotions); and Clarity (i.e., confusion about what one is feeling). Higher scores are associated with increased difficulties in emotion regulation.

#### Five-facet mindfulness questionnaire

The Five-Facet Mindfulness Questionnaire (FFMQ; Baer et al. 2006) is a 39-item self-report measure of mindfulness strategies in daily life, with higher scores indicating greater use of mindfulness. Items (e.g., “I perceive my thoughts and emotions without having to react to them,” “Even when I’m feeling terribly upset, I can find a way to put it into words”) were rated on a 5-point Likert scale (1 = never or rarely true, 5 = very often or always true). The FFMQ comprises five subscales (Cronbach’s *α*s ≥ 0.76) including (1) Acting with Awareness (i.e., remaining present in the moment), (2) Non-reacting (i.e., avoiding reflexive reactions to emotions), (3) Non-judging (i.e., non-judgmental attitude towards one’s emotions), (4) Observing (i.e., the tendency to pay attention to current emotional states), and (5) Describing (i.e., the ability to describe emotions in words).

### Procedure

Participants provided informed consent and were administered a structured clinical interview by a trained interviewer. Next, participants completed a series of experimental tasks not relevant to the present investigation followed by self-report questionnaires, which included the measures of emotion awareness and regulation strategies reported in the present study. Participants were then debriefed and compensated in cash ($10 per hour) for participation.

## Results

Demographic information and clinical characteristics are provided in Table 1. Groups did not differ on age, gender, ethnicity, or education. For the main hypotheses, we completed analyses both with and without current symptom covariates (IDSC and YMRS), noting where analyses differed with the inclusion of covariates.^2^ Effect sizes of mean differences are presented in the metric of Cohen’s *d*.

**Fig. 1:**
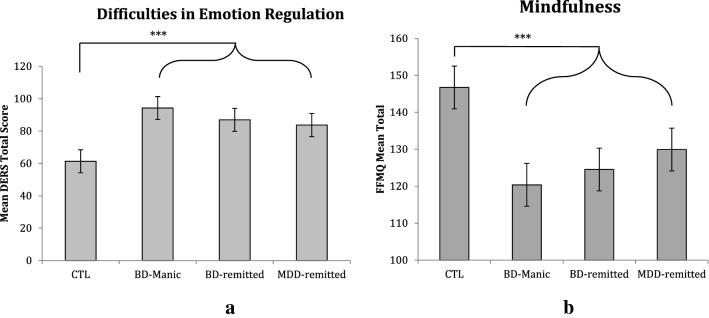
Group differences in total scores for difficulties in emotion regulation (**a**) and mindfulness-based regulation strategies (**b**)*.* Error bars represent standard errors of the mean. *p < .05, **p < .01, ***p < .001

Our first aim examined group differences in emotion regulation difficulties (based on the DERS total score). Consistent with our predictions, all three clinical groups reported greater difficulties in emotion regulation compared with the CTL group. As seen in Fig. 1a, for difficulties in emotion regulation, there was a main effect of Group (*F*(3, 108) = 14.18, *p* < 0.001). Post-hoc analyses confirmed that, relative to the CTL group (*M* = 61.33, *SD* = 15.61), the BD-remitted (*M* = 86.97, *SD* = 21.92, *d* = 1.35), MDD-remitted (*M* = 83.75, *SD* = 18.72, *d* = 1.30), and BD-manic (*M* = 94.28, *SD* = 21.79, *d* = 1.74) groups had significantly greater difficulties in emotion regulation (*p’*s < 0.001). No other group differences were significant (*p’*s ≥ 0.21). As seen in in Table 2, post-hoc analyses indicated that the three clinical groups differed from the CTL group (i.e., reported significantly greater emotion regulation difficulties) on the non-acceptance, goals, impulse, and strategies subscales of the DERS; as above, the clinical groups did not differ from one another. Furthermore, the BD-remitted and MDD-remitted groups also reported greater difficulties on the clarity subscale than the CTL group. No group differences emerged on the awareness subscale.

**Table 2:** Descriptive statistics for FFMQ and DERS by diagnostic group

	BD-remitted (*n* = 32)	BD-manic (*n* = 19)	MDD-remitted (*n* = 32)	CTL (*n* = 30)	Main effect of group
FFMQ					
Non-reactivity	20.72 (4.33)^a^	19.63 (4.68)^b^	21.28 (5.13)^c^	24.23 (3.99) ^a,b,c^	*F*(3, 109) = 4.99, *p* = .003
Observing	25.53 (7.00)	26.89 (9.00)	26.75 (7.53)	26.80 (13.72)	*F*(3, 109) = .35, *p* = .788
Acting with awareness	22.13 (5.89)^a^	20.12 (5.77)^b,f^	23.19 (5.58)^c,f^	30.00 (3.92)^a,b,c^	*F*(3, 109) = 17.63, *p* < .001
Describing	28.31 (6.78)^a^	27.95 (7.86)^b^	29.19 (5.57)^c^	33.53 (4.70)^a,b,c^	*F*(3, 109) = 4.96, *p* = .003
Non-Judging	27.84 (8.77)^a^	25.79 (8.16)^b^	28.90 (5.99)^c^	33.83 (5.49)^a,b,c^	*F*(3, 107) = 5.98, *p* = .001
DERS					
Non-acceptance	13.25 (5.25)^a^	14.67 (7.34)^b^	13.09 (4.90)^c^	9.90 (4.86)^a,b,c^	*F*(3, 108) = 3.54, *p* = .017
Goals	15.34 (4.29)^a^	16.67 (5.59)^b^	15.53 (4.47)^c^	10.80 (4.21)^a,b,c^	*F*(3, 108) = 8.87, *p* < .001
Impulse	12.69 (4.80)^a,d^	16.94 (7.22)^b,d,f^	12.03 (4.30)^f^	8.03 (2.51)^a,b,c^	*F*(3, 108) = 14.15, *p* < .001
Awareness	15.03 (5.00)	14.94 (4.72)	13.66 (4.04)	12.80 (4.43)	*F*(3, 108) = 1.58, *p* = .199
Strategies	18.66 (7.98)^a^	20.55 (7.09)^b^	18.56 (6.94)^c^	10.87 (3.38)^a,b,c^	*F*(3, 107) = 10.94, *p* < .001
Clarity	12.00 (5.09)^a^	10.28 (3.78)	11.09 (2.99)^c^	8.87 (3.78)^a,c^	*F*(3, 108) = 3.75, *p* = .013

*BD-remitted* Bipolar I disorder remitted group, *BD-manic* Bipolar I disorder manic group, *MDD* major depressive disorder remitted group, *CTL* healthy control group, *FFMQ* Five Facet Mindfulness Questionnaire (higher scores = increased mindfulness), *DERS* Difficulties in Emotion Regulation Scale (higher scores = more difficulties in emotion regulation). Mean scores are displayed with standard deviations in parentheses. In each row, means sharing a superscript letter (*a*, *b, c, d, or e*) were significantly different in pairwise group comparisons (*p* < .05): ^*a*^BD-remitted significantly different from CTL; ^b^BD-manic significantly different from CTL; ^c^MDD-remitted significantly different from CTL; ^d^BD-remitted significantly different from BD-manic; ^e^BD-remitted significantly different from MDD; ^f^BD-manic significantly different from MDD-remitted

Our second aim examined group differences in mindfulness-based regulation strategies based on the FFMQ total score. Consistent with our predictions, all three clinical groups reported decreased use of mindfulness compared to the CTL group. As seen in Fig. 1b, there was a main effect of Group (*F*(3, 107) = 10.47, *p* < 0.001) for trait mindfulness, which was characterized by significantly decreased mindfulness in the BD-manic (*M* = 120.37, *SD* = 20.98, *d* = 1.46), BD-Remitted (*M* = 124.53, *SD* = 19.36, *d* = 1.30), and MDD-Remitted (*M* = 129.93, *SD* = 19.97, *d* = 0.96) groups relative to the CTL group (*M* = 146.73, *SD* = 14.56, *p’*s < 0.001); however, the clinical groups did not differ from one another. As seen in in Table 2, post-hoc analyses of the FFMQ subscales indicated that the three clinical groups differed from the CTL group (i.e., scored significantly lower on) on the non-reactivity, acting with awareness, describing, and non-judging subscales of the FFMQ; as above, the clinical groups did not differ from one another. No group differences emerged on the observing subscale.

## Discussion

The present study investigated group differences in trait emotion regulation and mindfulness-based regulation strategies in a sample of adults with currently manic BD, remitted BD, remitted MDD, and a healthy non-psychiatric control group. Our results suggest that all three clinical groups reported greater difficulty regulating their emotions and used fewer mindfulness-based regulation strategies in their everyday lives compared with individuals without a psychiatric history. However, the three mood-disordered groups did not differ significantly from one another in either overall emotion regulation or mindfulness-based regulation strategies. Post-hoc examination of individual subscales generally suggested these patterns were consistent across distinct facets of emotion regulation difficulties and mindfulness. Together, these results indicate that BD and MDD are both characterized by increased levels of emotion regulation difficulties, as well as decreased mindfulness, compared to the healthy control group. Interestingly, this pattern seems to be transdiagnostic and consistent regardless of current mood state.

## Conclusions

This preliminary investigation sheds light on emerging evidence regarding patterns of emotion regulation strategies across mood disorders. First, this work is consistent with a growing body of work documenting increased emotion regulation difficulties (including emotion-relevant impulsivity), and decreased utilization of adaptive mindfulness-based strategies, in mood disorders (e.g., Gruber 2011; Gruber et al. 2013; Wolkenstein et al. 2014; Muhtadie et al. 2014; Rottenberg et al. 2005; Garnefski and Kraaij 2006; Joormann 2010; Gruber et al. 2014; Hanssen et al. 2019; Gilbert et al. 2013; Vine and Aldao 2014; Rheenen et al. 2015; Giovanelli et al. 2013; Park et al. 2014; Weinstock et al. 2018), and importantly extends this work by examining bipolar and unipolar mood-disordered groups within a single study and with the additional inclusion of currently hypomanic/manic bipolar individuals. Interestingly, post-hoc analyses exploring individual facets of emotion regulation and mindfulness-based strategies suggested that the emotion awareness subscales on both the DERS and FFMQ were not significantly different between groups. This suggests that mood-disordered individuals may not exhibit differences from non-psychiatric controls in their tendency to notice and pay attention to what they are feeling. This is surprising considering that, in the present investigation, such individuals self-reported difficulties with putting their feelings into words and endorsed an increased tendency to judge and react negatively to their own feelings. Further work is warranted to examine the extent to which intact awareness, as well as prior exposure to mindfulness-based strategies and interventions, may contribute to the etiology and course of mood symptomatology. Additional work in larger sample sizes using multivariate analytic approaches might also reveal unique versus common underlying factors across distinct types of emotion regulation strategies.

Some limitations of the present research design should be mentioned. First, we note that although the sample sizes were larger than those from previous studies on this topic and relatively large considering the severe nature of the psychiatric groups recruited, the present sample sizes were still somewhat small. Power analyses (using standard estimates of adequate power of 0.80) suggest that our sample size was adequately powered to detect a medium to large effect size (i.e., η_p_^2^ ≥ 0.11), but it is possible that we may have failed to detect more subtle effects. Future studies with larger sample sizes are an important next step to examine the generalizability of these findings. Second, although the current research identifies group differences in mindfulness and emotional regulation, without a longitudinal design or the inclusion of interventions aimed at modulating emotional awareness and regulation strategies, the current research is not designed to elucidate the etiological role of emotional awareness and regulation in the trajectory of BD and MDD. Third, while our study has provided first-step pilot data suggesting that emotion regulation difficulties and deceased mindfulness may be a more trait-like rather than state-dependent feature of mood disturbance, further work is necessary to examine underlying mechanisms that give rise to these observed patterns. Given our outpatient manic BD participants endorsed mild to moderate symptom severity, future work replicating these findings in acutely manic as well as currently depressed BD individuals would be informative. Fourth, the study relied on self-report measures and additional research obtaining converging results using behavioral and neurophysiological indices of emotion regulation is warranted (e.g., Miller and Chapman 2001). In addition, while this study provided valuable insights regarding self-reported measures of emotion regulation and mindfulness, it will be critical to examine how such findings translate to everyday life. An examination of state-specific regulatory patterns that target the regulation of specific positive and negative emotion states within BD and MDD would be a useful expansion of the current research. Finally, forthcoming research could explore the effectiveness of mindfulness training in reducing impulsivity and increasing emotional awareness in individuals with BD and MDD. Such work may help uncover the possible causal role that emotion regulation and mindfulness may play in the etiology and severity of affective disorders.

## Data Availability

Not applicable.

## Footnotes

1 Unless otherwise specified, parallel results emerged when running analyses with and without the smaller hypomanic subgroup excluded in the BD group.

2 We suggest that any results based on the inclusion of symptom scores as covariates be interpreted with caution given that controlling for current symptoms to minimize between-group variability violates important statistical assumptions (e.g., (Baer et al. 2006)). In addition it is theoretically questionable given a primary aim was to examine group differences as a function of symptom severity (i.e., comparing our manic BD group to remitted BD and MDD groups). Nonetheless we present these results for full transparency. Thus we did not control for current symptoms in the present analyses.
